# Efficacy and Safety of Intravenous Ferric Carboxymaltose in Patients with Postoperative Anemia Following Same-Day Bilateral Total Knee Arthroplasty: A Randomized Controlled Trial

**DOI:** 10.3390/jcm10071457

**Published:** 2021-04-02

**Authors:** Man Soo Kim, In Jun Koh, Keun Young Choi, Sung Cheol Yang, Yong In

**Affiliations:** 1 Department of Orthopaedic Surgery, Seoul St. Mary’s Hospital, College of Medicine, The Catholic University of Korea, 222, Banpo-daero, Seocho-gu, Seoul 06591, Korea; kms3779@naver.com (M.S.K.); heaxagon@hanmail.net (K.Y.C.); scillay@gmail.com (S.C.Y.); 2 Department of Orthopaedic Surgery, Eunpyeong St. Mary’s Hospital, College of Medicine, The Catholic University of Korea, 1021, Tongil Ro, Eunpyeong-gu, Seoul 03312, Korea; oskoh74@gmail.com

**Keywords:** total knee arthroplasty, bilateral, ferric carboxymaltose, hemoglobin anemia, response

## Abstract

(1) Background: The purpose of this study was to evaluate the efficacy and safety of intravenous (IV) ferric carboxymaltose (FCM) to treat acute postoperative anemia following same-day bilateral total knee arthroplasty (TKA). (2) Methods: A total of 118 patients who underwent same-day bilateral TKA were randomly assigned to two groups: an FCM group (FCM infusion, 58 patients) and a Control group (placebo with normal saline, 60 patients). The primary endpoint was the number of responders with a Hb increase of two or more points by the second postoperative week. The secondary endpoints were Hb level, iron metabolism variables and blood transfusion rate at 2, 6 and 12 weeks after surgery. (3) Results: The FCM group had more Hb responders than the Control group (62.1% vs. 31.6%, *p* < 0.001). The Hb level was significantly higher in the FCM group during 12 weeks after surgery (all *p* < 0.05). Ferritin, iron and transferrin saturation levels were significantly higher in the FCM group from 2 to 12 weeks postoperatively (all *p* < 0.05). There was no difference in transfusion rate after surgery (*p* > 0.05). (4) Conclusion: In patients with postoperative anemia after same-day bilateral TKA, IV FCM infusion significantly improved Hb response two weeks after surgery without severe adverse events compared to placebo. In contrast, transfusion rate and various parameters of quality of life assessment up to 12 weeks did not vary between these groups. Level of evidence: Level I.

## 1. Introduction

After major surgery, the incidence of postoperative anemia is almost 90% [1]. During total knee arthroplasty (TKA), the risk of intraoperative bleeding is small because pneumatic tourniquets are generally used. However, more than 80% of total blood loss occurs within 24 h of TKA [2]. In addition, hidden blood loss accounts for approximately 50% of total blood loss [3]. Anemia in the perioperative period causes overall physical deterioration, such as fatigue, dizziness, reduced exercise tolerance or delayed recovery [4]. It also increases morbidity and mortality and negatively affects quality of life (QOL) [5,6]. Perioperative anemia has been associated with increased hospital stay and incidence of postoperative complications [5,6].

Various studies on preoperative anemia have been conducted. It is recommended that iron supplementation is used to correct anemia a few weeks prior to surgery [5,6]. However, there are few studies that have addressed iron supplementation for postoperative anemia [7]. In addition, it is burdensome and not always possible for patients who are scheduled for surgery to visit the hospital before surgery so that anemia can be identified and treated [8]. Furthermore, it is not possible to accurately predict which patients will develop postoperative anemia after TKA [9].

Intravenous (IV) ferric carboxymaltose (FCM) is an innovative IV iron formulation and stable iron complex that consists of a ferric hydroxide core stabilized by a nondextran carbohydrate shell [10]. FCM can be administered in a single, rapid session (15 min infusion) at large doses (15 mg/kg; maximum of 1000 mg/infusion) [10]. It has the advantage of being low risk with few gastrointestinal side effects [7]. Some studies have investigated the effect of IV FCM in the postoperative period after TKA [7,11,12]. However, no prior randomized controlled trial (RCT) has evaluated IV FCM as a treatment for anemia during the perioperative period in patients undergoing same-day bilateral TKA [13]. The purpose of this study was to evaluate the efficacy of IV FCM to treat acute postoperative anemia following same-day bilateral TKA. It was hypothesized that IV FCM would be an effective and safe treatment for postoperative anemia in patients undergoing same-day bilateral TKA.

## 2. Methods

**Figure 1:**
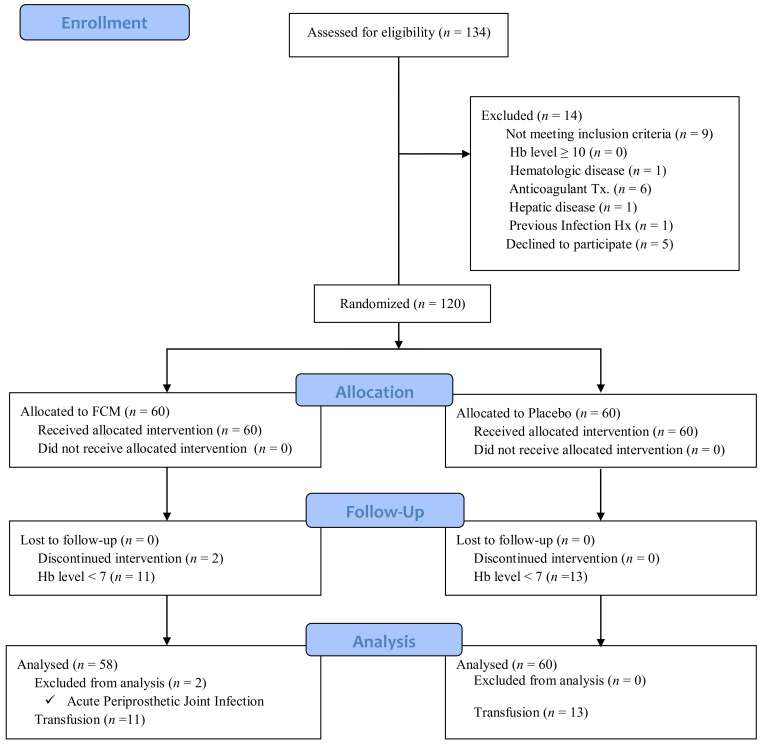
Flow of patient enrollment, randomization and follow-up.

A total of 134 cases of same-day bilateral TKAs were performed at our institution between March 2018 and May 2020. Among them, a prospective, randomized, patient-blinded, placebo-controlled study was performed on 120 patients with a Hb level <10 g/dL (from immediately after to 3 days after surgery). A total of 120 patients was randomly assigned to two groups. Fifty-eight patients were included in the FCM group (IV FCM infusion) after two patients were excluded due to periprosthetic joint infection and 60 patients in the Control group (Figure 1). This study was approved by the institutional review board at our hospital. All eligible subjects were informed of the standardized information on the clinical trial and provided written informed consent. The trial was registered with ClinicalTrials.gov (NCT03561480).

The study was conducted on patients with acute isovolemic anemia after same-day bilateral TKA. The definition of anemia is hemoglobin <12 g/dL in men or <13 g/dL in women. However, mild anemia was not included in this study [14]. Instead, we used Hb level <10 g/dL, which is a standard for moderate anemia, as the cut-off value [14]. The Hb level criterion used for transfusion is decreasing. In our study, the criterion for blood transfusion was strictly limited to cases with a Hb level <7 g/dL [15,16,17]. However, a Hb threshold of 8 g/dL was applied to patients with cardiovascular disease [18]. The preoperative exclusion criteria were hematologic disease, thromboembolic disease, hepatic or renal disease, coagulation disorder, infection, malignancy, anticoagulant therapy, hypersensitivity to iron and a history of a blood transfusion within the previous month, as well as an American Society of Anesthesiologists (ASA) grade of 3 or higher.

Randomization to receive FCM (FCM group) or placebo (Control group) was accomplished using the block randomization method to generate balanced groups. Randomization was performed using a method of sealed envelopes that contained information regarding procedure type. These envelopes were opened prior to the procedure. Because FCM is a typical dark brown color, both FCM and control treatments and IV lines were covered with black vinyl to ensure patient blinding.

The FCM group received 500 mg or 1000 mg of FCM, Ferinject^®^ (Vifor Pharma, Flughofstrasse, Switzerland), according to body weight (1000 mg for body weight ≥50 kg or 500 mg for body weight <50 kg). The Control group received 200 or 100 mL of normal saline depending on body weight (0.9% sodium chloride solution; 200 mL for bodyweight ≥50 kg or 100 mL for body weight <50 kg) [19]. FCM was injected as a single IV infusion mixed with 100 mL of normal saline or as an undiluted bolus injection over an injection time of 15 min for 1000 mg or 6 min for 500 mg. The FCM and normal saline were injected only once. An antifibrinolytic drug was not used in this study.

The primary endpoint was the ratio of Hb responders at the second postoperative week. The minimally clinically important difference (MCID) of Hb change is generally accepted to be 2 g/dL [20,21]. A Hb responder was defined by an increase in Hb by 2 g/dL or more compared to baseline [14,22]. The baseline Hb level was that when FCM or normal saline was administered after surgery. The secondary endpoints were Hb level; iron metabolism variables of ferritin concentration, serum iron, total iron-binding capacity (TIBC) and transferrin saturation levels; the number of patients requiring blood transfusion; Brief Pain Inventory (BPI) and QOL scale scores using EQ-5D at 2 and 6 weeks and 3 months after surgery.

The Brief Pain Inventory (BPI) is widely used to measure patient pain intensity and the amount of disability in the functional aspects of daily life due to pain [23]. There are two categories of BPI: pain interference and pain intensity. Pain interference is divided into two categories of activity interference and affective interference. Activity interference involves general activities and the physical aspects of everyday life such as walking. Affective interference includes the emotional or internal aspects of everyday life, such as enjoyment and/or mood. Pain intensity is measured in four categories of worst, least, on average in the last 24 h and current, while pain interference is measured in seven categories of mood, work, general activity, walking, relationships, enjoyment of life and sleep. Patients rate each of these parameters on a scale of 0–10, where 10 means severe exacerbation of pain intensity and full interference to daily life. In the case of BPI pain intensity, the MCID is defined as a pain score change of 2.1–2.2 [24]. The MCID achievement of BPI pain intensity was also evaluated.

The EQ-5D is a commonly used and validated measurement tool for health-related QOL. It was developed by the EuroQol (EuroQ of Life) group and consists of five parts of mobility, self-care, daily activities, pain/discomfort and anxiety/depression. Each sector is measured using three grades of no problems, some problems, and extreme problems [25].

Safety and tolerability were continuously monitored during the three-month follow-up period. Symptoms and discomfort after FCM or saline injection were recorded. The side effects that occurred during the follow-up period were treated at the physician’s discretion. We also investigated whether there was a difference in length of hospital stay between the two groups.

### Statistical Analysis

The target sample size was calculated for each group at a power of 80% to minimize the probability of a type II error. The alpha level of significance was set at 0.05. Because the MCID of Hb change was defined as 2 g/dL, the Hb responder was set to a change of Hb level of 2 g/dL or more [20,21]. The proportion of Hb responders 2 weeks after surgery was set as the primary outcome. The clinically meaningful important difference between Hb responders in the FCM and Placebo groups was set to 30%, in reference to the results of a previous study [22]. The estimated sample size was 44 patients for each group. Sixty patients were needed per group based on an estimate of 25% follow-up loss. Continuous variables were analyzed using a *t*-test, while categorical data were analyzed using a Chi-square test or Fisher’s exact test where appropriate for two independent samples. Descriptive analyses were based on frequency and percentage for categorical variables and mean and standard deviation for continuous variables. Hematologic markers including Hb and iron metabolism variables were assessed by repeated-measures analysis of variance test (ANOVA) and Bonferroni post hoc test to discover which specific means differed from one another. All statistical analyses were performed using SPSS ver. 21.0 program (SPSS Inc., Chicago, IL, USA). Values of *p* < 0.05 were considered statistically significant. Statistical analysis was performed from March 2018 to May 2020.

## 3. Results

**Figure 2:**
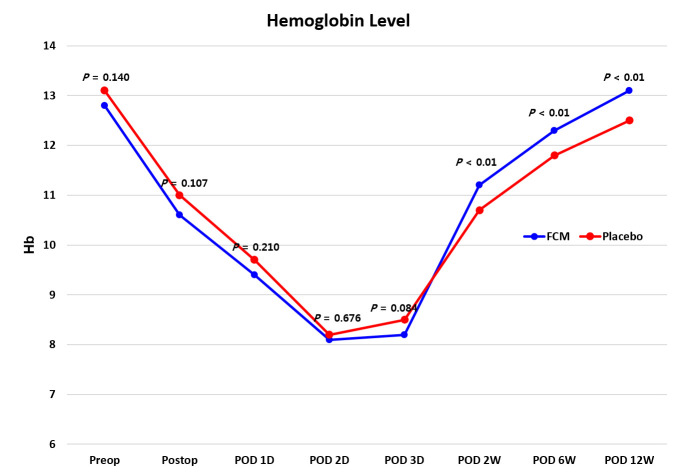
Hemoglobin level from preoperative to postoperative 12 weeks.

There were 58 patients in the FCM group and 60 patients in the control group. There were no differences in demographic and hematologic laboratory findings between the two groups (Table 1). The postoperative Hb level did not vary between the two groups from immediately after surgery to three days postoperative (Figure 2) Two weeks after surgery, the FCM group had more Hb responders than the Control group (62.1% vs. 31.6%, *p* < 0.001). The Hb level was significantly improved at 2, 6 and 12 weeks after surgery compared to the postoperative baseline in both groups. Patients in the FCM group recovered preoperative Hb level at 12 weeks after surgery. However, patients in the Control group did not recover until 12 weeks postoperatively (Figure 2). After IV FCM was administered, Hb level was significantly higher in the FCM group than in the Control group at 2, 6 and 12 weeks after surgery (Figure 2, Table 2). Serum ferritin, iron and transferrin saturation was not significantly different between the two groups before surgery. However, these values were significantly higher in the FCM group than the Control group 2, 6 and 12 weeks after surgery (Table 2). There was no difference in TIBC level between the two groups before surgery; however, the Control group had a higher TIBC level than the FCM group until 12 weeks after surgery (Table 2).

**Figure 3:**
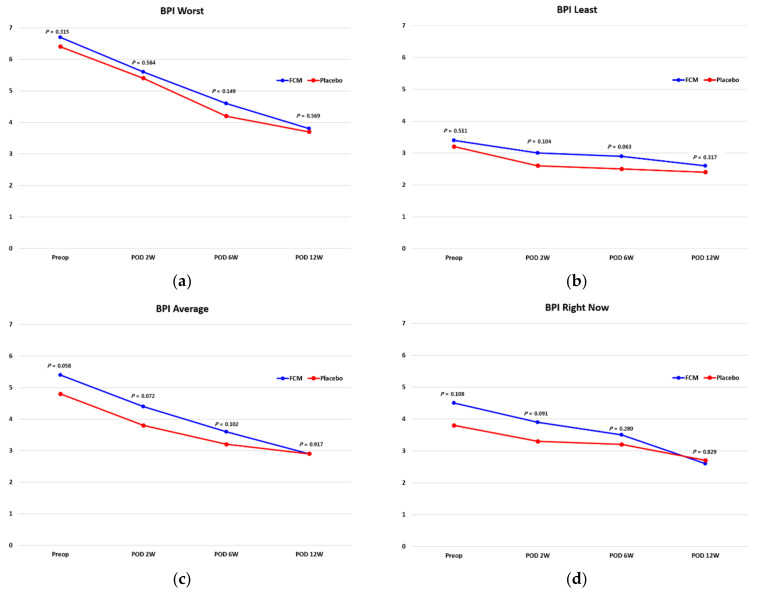
Brief Pain Inventory (BPI) and pain intensity. Worst (**a**), least (**b**), average (**c**) and right now (**d**).

The QOL measured by EQ 5D did not show any differences between the two groups preoperatively or at 2, 6 or 12 weeks postoperatively (Table 3). The BPI pain and BPI interference were similar between the two groups during the follow-up period (Table 4 and Figure 3). There was no significant difference in the achievement of MCID for BPI worst, least, average and right now pain levels between groups (all *p* > 0.05). The hospitalization period was not different between the two groups, with the FCM group being 7.2 days and the Placebo group 7.6 days (*p* > 0.05). Transfusion was performed within 3 days after surgery during the 12-week follow-up period. The Hb level at the time of transfusion was 6.6 ± 0.2 g/dL, and the median value was 6.7 g/dL. There was no significant difference between the FCM group and the Control group (11 cases, 19.3% vs. 13 cases, 21.7% respectively, *p* = 0.716).

There were three adverse events across both groups. Two adverse events of pruritus and headache occurred in the FCM group, while phlebitis occurred once in the Control group. No wound complications occurred in either group. All adverse events resolved uneventfully. Neither group had serious complications that required additional treatment (*p* > 0.05).

## 4. Discussion

The most important finding of this study was that postoperative IV FCM infusion significantly increased the proportion of Hb responders at two weeks after surgery. In addition, Hb level significantly increased in the FCM group compared to those in the Control group up to 12 weeks after surgery. Iron-related factors were also significantly higher in the Control group up to 12 weeks after surgery.

Various studies have been conducted on perioperative anemia in arthroplasty surgery [26,27]. There is interest in anemia after arthroplasty because it is mostly performed in the elderly and is associated with perioperative bleeding. However, most prior studies have focused on reducing transfusion requirements after surgery through the treatment of preoperative anemia. In addition, various guidelines have focused on improving clinical aspects through the correction of preoperative anemia [26,27]. Because most of the bleeding after arthroplasty occurs during the perioperative period [2,3], it is essential to know the effect of treatment on anemia during this period. However, although TKA is widely performed, there are little data regarding the treatment of postoperative anemia after TKA [7,8,11,12]. In particular, no studies have investigated the effect of IV FCM infusion in this setting, which is known to be an effective and stable option for iron deficiency anemia treatment. Therefore, a strength of this study is that it used a double-blind placebo-controlled RCT method to demonstrate the effect of FCM in same-day bilateral TKA.

This study was performed based on a Hb response threshold of 2 g/dL or more compared to baseline at two weeks after surgery. A 2 g/dL increase in Hb was set as a meaningful value [14,19,22]. Although Hb response is used as an important index to judge the effect of iron supplementation as a treatment for anemia, it has not been used in TKA-related studies [14,19,22]. One study used an increase in Hb level of 2 g/dL or more as a Hb response to examine the effect of IV FCM after surgery in another clinical setting [19,22]. In this study, there was a significantly higher percentage of Hb responders in the FCM group (62%) than in the placebo group (31%) [22]. In addition, the FCM group showed a significantly higher Hb level between 2 and 12 weeks postoperatively than the Control group. The FCM group also showed significantly superior results of all iron metabolism variables for up to three months.

If postoperative anemia persists, it can affect postoperative recovery and QOL [5,6]. Therefore, QOL and functional recovery after surgery were verified using the EQ 5D and BPI, There was no difference in QOL or BPI at 2, 6 or 12 weeks after surgery in the two groups. Most patients undergoing bilateral TKA are elderly and have a variety of comorbidities. However, a 12-week recovery period after bilateral TKA might be unable to adequately assess QOL. In fact, several studies also did not show differences between the FCM and control groups in QOL within a period of fewer than 12 weeks in elderly patients [11,22]. Therefore, a comparison of QOL between the two groups should be confirmed through large, longer-term studies [11].

In this study, there was no difference in the rate of transfusion between the two groups. The role of treatment for preoperative or postoperative anemia is important to reduce the transfusion rate after surgery [28]. In this study, IV FCM was administered when the Hb level was reduced to 10 g/dL or less following bilateral TKA. Transfusion was only performed between one and three days after surgery in both groups. In all cases of transfusion, IV FCM and placebo were administered before transfusion. In transfused patients with Hb of 7 g/dL or less, a large amount of blood loss after bilateral TKA occurred in the acute phase within three days after surgery. Therefore, this blood loss is not an appropriate outcome to accurately identify the effect of IV FCM because of the insufficient time for effective production [29]. The time to effect of IV FCM in practice is approximately one week [29,30]. Several studies comparing transfusion rates for postoperative anemia also showed no difference in transfusion rate [7,8].

Various side effects of IV iron supplementation have been reported. Prior reports have suggested that IV iron increases the risks of cardiovascular events [31], infection rates [32,33], and life-threatening hypersensitivity [34]. However, in this study, hypersensitivity, cardiovascular events and infection were not reported in either group. In addition, there was no difference in the proportion of side effects between the two groups. In the FCM group, pruritus appeared in one patient, and headache in another patient. One patient in the Control group developed phlebitis. IV FCM is a safe drug that is not associated with an increased risk of severe adverse events or infections [29,35], supporting the results of our study. The two FCM patients with side effects recovered without further abnormality.

Perioperative blood loss leads to a Hb decrease after surgery [28]. Inflammation induced by surgery also affects iron metabolism [36]. Hepcidin is a master regulator of iron metabolism that prevents the release of intracellular iron stores, inhibits iron uptake from the gut and reduces iron excretion into the blood [36,37]. Hepcidin is increased by inflammatory cytokines such as interleukin 6 [38]. This inflammatory reaction leads to functional iron deficiency anemia and consequently leads to iron-restricted erythropoiesis. After the administration of iron, reticulocytosis with erythropoiesis occurs to correct iron deficiency anemia [39]. IV FCM increases the possibility of using iron by providing sufficient iron refill [40] and increasing circulating iron [41]. Iron supplementation inhibits the production of TNF-a and promotes the recovery of hemoglobin [38].

This study has several limitations. First, most of the patients were female, and the study was conducted in one research institute. All patients in the current study were of Asian ethnicity. It is well known that a high proportion of OA in Asian patients occurs in women [42,43,44,45]. Given that this study was limited to Asian patients from one site, care must be taken when generalizing these results. Second, transfusion may be a confounding factor in determining the effect of FCM. All patients who underwent bilateral TKA and had a Hb level of 10 g/dL or less within three days after surgery were included. Blood transfusion was performed after IV FCM or placebo administration if baseline Hb was less than 7 g/dL. Therefore, changes in blood-related factors due to transfusion clearly could have interfered with the comparison of IV FCM and placebo groups. However, there was no difference in the rate of transfusion between the two groups. Third, the iron metabolism variable was measured three times after surgery. If these measurements also were performed on postoperative days 1–3, a clearer and more detailed longitudinal comparison of iron metabolism would have been possible between the two groups. However, we did not add this test because the enrolled subjects were targeted based on Hb level less than 10 g/dL after surgery and moderate isovolemic anemia [22]. Fourth, the economic effect of IV FCM was not considered in this study. Some studies have investigated the economic effects of IV FCM [46,47]. However, because the comparison of economic effects is determined according to the reimbursement policy of medical insurance in each country, it is difficult to compare clear cost effectiveness [46,47]. A longer follow-up of the effectiveness of FCM can give a clearer answer to the results of this study. Finally, it is difficult to confirm the safety of FCM and a lack of adverse events based on a study of only 60 patients. Although FCM has been approved for use in iron deficiency anemia patients, we plan to evaluate safety through a study with a larger number of patients. Despite these limitations, this study has a great advantage in being the first to compare the effect of postoperative IV FCM with placebo through prospective RCT in patients undergoing same-day bilateral TKA with a large amount of bleeding.

## 5. Conclusions

In patients with postoperative anemia after same-day bilateral TKA, IV FCM significantly improved Hb response at two weeks after surgery without significant adverse events compared to placebo. In contrast, transfusion rates and various parameters of QOL assessment up to 12 weeks did not vary between these two groups.

## Figures and Tables

**Table 1:** Baseline patient characteristics.

Characteristics	Ferric Carboxymaltose (*n* = 58)	Placebo (*n* = 60)	*p*-Value
**Age, mean (SD), y**	69.2 (4.7)	70.3 (4.1)	0.169
**Sex**			0.819
**Male**	6 (10.3%)	7 (11.7%)	
**Female**	52 (89.7%)	53 (88.3%)	
**Body Mass Index, mean (SD)**	26.5 (3.8)	27.2 (3.2)	0.253
**Comorbidities**			
**Hypertension**	34 (58.6%)	31 (51.7%)	0.448
**Diabetes**	12 (20.7%)	8 (13.8%)	0.287
**Cardiovascular**	4 (6.9%)	5 (8.3%)	0.769
**Brain**	0 (0%)	3 (5.0%)	0.085
**Thyroid**	3 (5.2%)	1 (1.7%)	0.293
**Kidney**	1 (1.7%)	1 (1.7%)	0.981
**Lung**	4 (6.9%)	2 (3.3%)	0.378
**Liver**	0 (0%)	1 (1.7%)	0.323
**Smoking**	0 (0%)	1 (1.7%)	0.323
**Alcohol**	1 (1.7%)	0 (0%)	0.307
**ASA**			0.082
**1**	18 (31.0%)	28 (46.7%)	
**2**	40 (69.0%)	32 (53.3%)	
**Torniquet time Right, mean (SD), min**	41.2 (5.4)	41.1 (6.3)	0.926
**Torniquet time Left, mean (SD), min**	42.0 (6.5)	41.7 (6.4)	0.822
**Total Hemovac amount, mean (SD), mL**	811.5 (435.4)	823.0 (348.8)	0.878
**Total blood loss during operation, mean (SD), mL**	369.7 (81.6)	391.0 (85.2)	0.168

Abbreviations: ASA, American Society of Anesthesiologists.

**Table 2:** Hemoglobin and iron metabolism variables by group.

Hematologic Markers	Ferric Carboxymaltose (*n* = 58)	Placebo (*n* = 60)	*p*-Value
**Hemoglobin, g/dL**			
Preoperative	12.8 (1.1)	13.1 (1.3)	0.140
Postoperative 2 weeks	11.2 (0.9)	10.7 (1.0)	0.007
Postoperative 6 weeks	12.3 (0.8)	11.8 (0.9)	0.004
Postoperative 12 weeks	13.1 (0.8)	12.5 (1.1)	0.007
**Serum Ferritin, ng/mL**			
Preoperative	108.6 (72.1)	115.0 (73.5)	0.641
Postoperative 2 weeks	1191.8 (491.1)	271.1 (226.6)	<0.001
Postoperative 6 weeks	753.4 (308.3)	169.9 (145.9)	<0.001
Postoperative 12 weeks	475.0 (231.1)	101.8 (98.2)	<0.001
**Iron, mcg/dL**			
Preoperative	80.0 (32.5)	82.6 (32.0)	0.667
Postoperative 2 weeks	86.8 (32.2)	56.5 (22.7)	<0.001
Postoperative 6 weeks	80.0 (24.0)	59.0 (23.3)	<0.001
Postoperative 12 weeks	83.4 (25.3)	60.7 (21.7)	<0.001
**Total Iron-Binding Capacity, mcg/dL**			
Preoperative	318.1 (46.9)	317.6 (60.2)	0.961
Postoperative 2 weeks	269.2 (39.2)	292.6 (44.8)	0.004
Postoperative 6 weeks	250.8 (34.6)	306.0 (54.7)	<0.001
Postoperative 12 weeks	255.4 (33.5)	320.3 (57.6)	<0.001
**Transferrin Saturation, %**			
Preoperative	25.6 (11.2)	27.1 (10.8)	0.497
Postoperative 2 weeks	32.7 (12.8)	19.9 (8.6)	<0.001
Postoperative 6 weeks	32.0 (9.6)	19.9 (8.4)	<0.001
Postoperative 12 weeks	32.4 (9.0)	19.9 (8.8)	<0.001

**Table 3:** Quality of life assessment using the EQ 5D.

EQ-5D	Ferric Carboxymaltose (*n* = 58)	Placebo (*n* = 60)	*p*-Value
**EQ 5D-1 Mobility**			
Preoperative	1.9 (0.4)	1.9 (0.4)	0.834
Postoperative 2 weeks	2.0 (0.5)	1.9 (0.4)	0.680
Postoperative 6 weeks	1.8 (0.4)	1.8 (0.4)	0.451
Postoperative 12 weeks	1.7 (0.5)	1.6 (0.5)	0.482
**EQ 5D-2 Self-Care**			
Preoperative	1.5 (0.6)	1.4 (0.6)	0.830
Postoperative 2 weeks	1.9 (0.6)	1.8 (0.5)	0.884
Postoperative 6 weeks	1.8 (0.4)	1.8 (0.5)	0.775
Postoperative 12 weeks	1.5 (0.6)	1.6 (0.6)	0.449
**EQ 5D-3 Usual Activities**			
Preoperative	1.7 (0.5)	1.7 (0.6)	0.903
Postoperative 2 weeks	2.1 (0.4)	2.1 (0.5)	0.982
Postoperative 6 weeks	1.8 (0.4)	1.8 (0.5)	0.528
Postoperative 12 weeks	1.6 (0.6)	1.7 (0.7)	0.564
**EQ 5D-4 Pain/Discomfort**			
Preoperative	2.2 (0.6)	2.2 (0.0)	0.418
Postoperative 2 weeks	2.0 (0.3)	1.9 (0.3)	0.750
Postoperative 6 weeks	1.9 (0.5)	2.0 (0.6)	0.309
Postoperative 12 weeks	1.6 (0.6)	1.4 (0.5)	0.076
**EQ 5D-5 Anxiety/Depression**			
Preoperative	1.5 (0.6)	1.4 (0.5)	0.453
Postoperative 2 weeks	1.5 (0.5)	1.5 (0.5)	0.778
Postoperative 6 weeks	1.5 (0.5)	1.5 (0.5)	0.628
Postoperative 12 weeks	1.7 (0.5)	1.7 (0.5)	0.616

**Table 4:** Brief Pain Inventory (BPI) and pain interference.

BPI Interference	Ferric Carboxymaltose (*n* = 58)	Placebo (*n* = 60)	*p*-Value
**BPI Interference General Activity**			
Preoperative	6.4 (2.6)	6.2 (1.8)	0.639
Postoperative 2 weeks	6.5 (1.8)	5.9 (1.8)	0.150
Postoperative 6 weeks	5.1 (1.5)	4.7 (1.1)	0.157
Postoperative 12 weeks	4.2 (1.3)	4.1 (1.1)	0.689
**BPI Interference Walking**			
Preoperative	6.0 (2.6)	6.0 (1.9)	0.868
Postoperative 2 weeks	6.1 (1.9)	5.9 (1.9)	0.645
Postoperative 6 weeks	5.3 (1.5)	4.8 (1.3)	0.125
Postoperative 12 weeks	4.5 (1.5)	4.2 (1.4)	0.456
**BPI Interference Work**			
Preoperative	6.3 (2.1)	6.1 (1.9)	0.695
Postoperative 2 weeks	6.2 (2.1)	6.0 (1.8)	0.522
Postoperative 6 weeks	5.3 (1.5)	4.8 (1.4)	0.108
Postoperative 12 weeks	4.4 (1.4)	4.3 (1.3)	0.735
**BPI Interference Sleep**			
Preoperative	5.5 (2.7)	5.2 (2.3)	0.498
Postoperative 2 weeks	5.5 (2.2)	5.1 (2.1)	0.340
Postoperative 6 weeks	4.6 (1.6)	4.1 (1.4)	0.098
Postoperative 12 weeks	3.9 (1.4)	3.8 (1.4)	0.991
**BPI Interference Relation**			
Preoperative	5.3 (2.8)	5.0 (2.5)	0.557
Postoperative 2 weeks	5.1 (2.6)	4.9 (2.2)	0.659
Postoperative 6 weeks	4.3 (1.9)	4.1 (1.7)	0.593
Postoperative 12 weeks	3.5 (1.5)	3.8 (1.6)	0.439
**BPI Interference Enjoyment**			
Preoperative	5.7 (2.5)	5.6 (2.1)	0.810
Postoperative 2 weeks	5.7 (2.1)	5.5 (1.9)	0.697
Postoperative 6 weeks	4.9 (1.3)	4.7 (1.5)	0.517
Postoperative 12 weeks	4.0 (1.3)	4.2 (1.4)	0.591
**BPI Interference Mood**			
Preoperative	5.8 (2.8)	5.4 (2.2)	0.488
Postoperative 2 weeks	6.0 (2.3)	5.5 (2.1)	0.284
Postoperative 6 weeks	5.0 (2.0)	4.4 (1.4)	0.084
Postoperative 12 weeks	4.3 (1.7)	4.0 (1.4)	0.366

## Data Availability

Data collected for this study, including individual patient data, will not be made available.
